# *In vivo* protein quality of selected cereal-based staple foods enriched with soybean proteins

**DOI:** 10.3402/fnr.v60.31382

**Published:** 2016-10-19

**Authors:** Laura Acevedo-Pacheco, Sergio O. Serna-Saldívar

**Affiliations:** Centro de Biotecnología-FEMSA, Escuela de Ingeniería y Ciencias, Tecnológico de Monterrey, Monterrey, México

**Keywords:** soybean proteins, maize tortillas, wheat flour tortillas, bread, PDCAAS, protein efficiency ratio, nitrogen retention

## Abstract

**Background:**

One way to diminish protein malnutrition in children is by enriching cereal-based flours for the manufacturing of maize tortillas, wheat flour tortillas, and yeast-leavened breads, which are widely consumed among low socio-economic groups.

**Objective:**

The aim was to determine and compare the essential amino acid (EAA) scores, protein digestibility corrected amino acid scores (PDCAAS), and *in vivo* protein quality (protein digestibility, protein efficiency ratio (PER), biological values (BV), and net protein utilization (NPU) values) of regular versus soybean-fortified maize tortillas, yeast-leavened bread, and wheat flour tortillas.

**Design:**

To comparatively assess differences in protein quality among maize tortillas, wheat flour tortillas, and yeast-leavened breads, EAA compositions and *in vivo* studies with weanling rats were performed. The experimental diets based on regular or soybean-fortified food products were compared with a casein-based diet. Food intake, weight gains, PER, dry matter and protein digestibility, BV, NPU, and PDCAAS were assessed. The soybean-fortified tortillas contained 6% of defatted soybean flour, whereas the yeast-leavened bread flour contained 4.5% of soybean concentrate.

**Results:**

The soybean-fortified tortillas and bread contained higher amounts of lysine and tryptophan, which improved their EAA scores and PDCAAS. Rats fed diets based on soybean-fortified maize or wheat tortillas gained considerably more weight and had better BV and NPU values compared with counterparts fed with respective regular products. As a result, fortified maize tortillas and wheat flour tortillas improved PER from 0.73 to 1.64 and 0.69 to 1.77, respectively. The PER improvement was not as evident in rats fed the enriched yeast-leavened bread because the formulation contained sugar that decreased lysine availability possibly to Maillard reactions.

**Conclusions:**

The proposed enrichment of cereal-based foods with soybean proteins greatly improved PDCAAS, animal growth, nitrogen retention, and PER primarily in both maize and wheat flour tortillas. Therefore, these foods can help to diminish protein malnutrition among children who greatly depend on cereals as the main protein dietary source.

Malnutrition could be solved globally by enrichment and fortification of food products with high-quality proteins and micronutrients (1, 2). According to Food and Agriculture Organization (FAO), 11.3% of the global population (805 million) were chronically malnourished in 2012–2014 (3). The easiest way to assist low socio-economic groups is to enrich flours at mill level with low-cost protein supplements that complement their essential amino acid (EAA) scores. The World Health Organization (WHO) estimated that in 2013, 98.9 million children younger than age 5 were underweight, from which 97.9 million inhabited developing countries (4).

It has been proposed that a solution to this problem is to increase the amount of protein in cereal staple foods (wheat, rice, and maize). These cereals provide most of the calories (more than 60%) and protein (about 50%) consumed in the average diet. Therefore, these staples are ideally suited for nutritional enrichment (5–7). In 2013, world production of all cereals was approximately 2,781 million tons. The FAO states that wheat, rice, and maize constitute more than 80% of the global cereal production (8). In Mexico, maize tortillas, wheat flour tortillas, and table bread are the basic cereal foods of the diet especially among low socio-economic groups. Unfortunately, maize-based tortillas and wheat-based bakery items lack the EAA lysine as well as optimum levels of relevant micronutrients such as iron, zinc, and vitamins A, D, E, and B12 (6, 7, 9).

Soybean (*Glycine max*) is the main legume produced worldwide. In 2013, more than 276 million tons were globally produced (8). Soybeans are a rich source of high-quality protein similar in terms of protein digestibility corrected amino acid scores (PDCAAS) to meat and dairy proteins. In addition, soybean products contain micronutrients and bioactive compounds such as isoflavones, flavonoids, saponins, both soluble and insoluble dietary fiber, and folic acid (10, 11). The industrial manufacturing of soybeans yields important products such as full-fat meals, oil, defatted meals, texturized proteins, concentrates, isolates, and soybean milk. The different array of soybean protein products contain from 40 to 90% protein with a good EAA score (12, 13). More importantly, soybean proteins provide high amounts of EAA that are found in lower concentrations in most cereals, making these proteins ideal sources to fortify these foods (14). From the processing point of view, different soybean proteins possess a range of functional properties such as gelling, emulsifying, foaming, water, and oil holding capacities (15).

Previous studies have shown that wheat bread, maize tortillas, and wheat flour tortillas can be successfully fortified with different soybean proteins without affecting sensory acceptance. These products contained 23–30% more protein compared to regular counterparts as well as an improved EAA score (9, 16, 17). To the best of our knowledge, there are no studies evaluating *in vivo* protein quality of soybean-fortified maize and wheat flour tortillas and bread at the same time. Thus, the aim of this research was to determine and compare the *in vivo* protein quality (animal growth and nitrogen retention) and the EAA scores of regular maize tortillas, yeast-leavened bread, and wheat flour tortillas compared with counterparts fortified with soybean proteins. Weanling rats were used because they have similar EAA requirements to infants and they are considered as a good model for protein quality studies (18).

## Materials and methods

### Chemical analyses of food products and diets

Compositions of animal-formulated diets are shown in Table 1. Foods were chemically characterized for moisture, protein, ether extract, crude fiber, and ash with the American Association of Cereal Chemists International (AACC) approved methods, respectively (44-15.02, 46-13.01, 30-20.01, 32-10.01, 08-01.01) (19). The nitrogen-free extract (NFE) was calculated by difference, whereas the energy values of regular and soybean-fortified foods were determined according to the Atwater coefficient by multiplying the NFE and protein content by 4 kcal/g and ether extract by 9.

**Table 1:** Formulation of regular and soybean-fortified cereal-based diets offered to weanling rats during the animal studies^1^

	Casein	Maize tortillas		Yeast-leavened bread		Wheat flour tortillas	
				
Ingredient (g/100 g)	CSD	MTD	SMTD	BRD	SBRD	WTD	SWTD
Corn oil	8.00	7.62	7.06	5.47	6.13	0	3.84
Water	0	0.29	1.55	0.62	0	2.97	2.19
Minerals AIN-76	3.50	3.50	3.50	3.50	3.50	3.50	3.50
Vitamins AIN-76	1.00	1.00	1.00	1.00	1.00	1.00	1.00
Cellulose	1.00	0	0	0.07	0.12	0.50	0.55
Casein	11.60	–	–	–	–	–	–
Starch	64.90	–	8.84	14.86	30.00	3.12	27.88
Sucrose	10.00	–	–	–	–	–	–
Maize tortillas	–	87.59	–	–	–	–	–
Soybean-fortified maize tortillas	–	–	78.05	–	–	–	–
Yeast-leavened bread	–	–	–	74.48	–	–	–
Soybean-fortified bread	–	–	–	–	59.25	–	–
Wheat flour tortillas	–	–	–	–	–	88.91	–
Soybean-fortified wheat flour tortillas	–	–	–	–	–	–	61.04

1 CSD, casein control diet; MTD, maize tortillas diet; SMTD, soybean-fortified maize tortillas diet; BRD, yeast-leavened bread; SBRD, soybean-fortified bread; WTD, wheat flour tortillas; SWTD, soybean-fortified wheat tortillas. Diets were formulated according to the PER protocol to contain 10% crude protein, 8% crude fat, and 1% crude fiber. In addition, they were supplemented with 1% vitamins and 3.5% mineral premix.

### Amino acid score and PDCAAS of food products

The complete EAA scores of regular and fortified tortillas and yeast-leavened breads were obtained following the Association of Official Analytical Chemists (AOAC) Official Method 982.30 E(a) (20). Briefly, the amino acid composition of all foods was determined by HPLC system (1525 binary pump, waters, Milford MA, USA) equipped with a 3.9×150 mm AccQ-Taq C18 column bonded with endcapped octadecylsilane, a fluorescence detector (Waters 2475 multi fluorescence), and an autosampler (Waters 717 plus). The concentration of particular amino acids was calculated by comparing with standards. The PDCAAS were calculated from the limiting EAA related to the requirement for children multiplied by the *in vivo* protein digestibility data estimated with weanling rats (21). The PDCAAS is recognized by the FAO/WHO and the US Food and Drug Administration as the preferred quick method for the estimation of protein quality.

### Preparation of diets

Maize flour tortillas and wheat flour tortillas were manufactured with composite flours containing approximately 6.0% of defatted soybean meal with a protein dispersibility index (PDI) of 23.2±0.11, whereas the yeast-leavened table bread with a bread flour containing 4.5% soybean protein concentrate with a PDI of 52±0.5. The preparation and characteristics of these regular and soybean-fortified cereal-based foods are detailed by Chuck-Hernández et al., Perez-Carrillo et al., and Lazo-Velez et al. (9, 16, 17). The regular and soybean-fortified products were dehydrated in a convention oven set at 50°C for 8 h and then ground in a Wiley mill equipped with a 2 mm sieve. The ground products were chemically characterized before diet formulation. Diets were formulated to contain 10% protein (1.6% nitrogen) according to the official PER AOAC 960.48 method (20). Diets were blended for 10 min in a Hobart mixer equipped with a paddle with the starch, vegetable oil, mineral premix (AIN 76), vitamin premix (AIN 76), and cellulose in order to contain equal amounts of corn oil, calories, and fiber. The control diet had similar composition to the experimental diets and contained casein (AIN high nitrogen) as the only source of protein. Diets were packed in sealed plastic bags and stored in a freezer (−20°C) until the beginning of the animal study and then transferred to a refrigerator (5°C).

### Animal study design and protein efficiency ratio bioassay

Assessment of the protein efficiency ratio (PER) was performed according to the official procedures recommended by the AOAC Official Method 960.48 (20). The protocol for this study (2013-Re-009) was approved by the Tecnológico de Monterrey Animal Care Committee and is based on international standards. Fifty-six weanling male Wistar rats (Bioinvert-Mexico) of approximately 21 days of age were housed in individual metabolic cages at a temperature of 22°C and a 12:12 light–dark cycle. They were acclimated to the new environment for 2 days. Rats were distributed to eight blocks according to their initial body weight. Each block had seven animals assigned to the seven different treatments. A total of 56 animals were used (8 blocks and 7 treatments) and fed *ad libitum* for 28 days. The control diet (CSD) contained casein as the source of protein, whereas the six experimental diets included regular or soybean-fortified tortillas or yeast-leavened bread. The experimental diets were named as maize tortilla diet (MTD), wheat flour tortillas diet (WTD), yeast-leavened bread diet (BRD), soybean-fortified maize tortillas diet (SMTD), soybean-fortified wheat flour tortillas diet (SWTD), and soybean-fortified yeast-leavened bread diet (SBRD) (Table 1). Food intake and body weights were measured every 2 weeks for the duration of the study. Experimental and adjusted PER values were calculated using the following equations: Experimental PER=weight gain/protein intake and adjusted PER=(2.5/Experimental PER value of the control casein)×(Experimental PER values of the respective treatment).

### Animal study for determination of nitrogen retention

Feces and urine were collected daily from day 14 until day 28 and immediately placed in a freezer at a temperature of −20°C. Food intake was carefully monitored for this period of time. In order to prevent urine nitrogen losses due to microbial contamination, urine containers were supplemented daily with 1 ml HCl (1:1). After 14 consecutive days of feces and urine collection, nitrogen was analyzed in feed, dried feces and urine following the AACC Official Kjeldahl Method 46–13:01 (19). Apparent protein digestibility, biological value (BV) and net protein utilization (NPU) were calculated using the following equations: % Protein digestibility=[[(Food intake) (% Nitrogen in Food)] – [(Feces) (% Nitrogen in Feces)]/[(Food intake) (% Nitrogen in Food)]] × 100, % BV=[[(Food intake) (% Nitrogen in Food)] – [(Feces) (% Nitrogen in Feces) – [(Urine)×(% Nitrogen in Urine)]]/[[(Food intake) (% Nitrogen in Food)] – [(Feces) (%Nitrogen in Feces)]]×100 and % NPU Value=[(% Protein Digestibility)×(% BV)]/100.

### Statistical analyses

Body weight, food intake, protein intake, PER, dry matter digestibility, protein digestibility, BV, and NPU values were analyzed using analysis of variance (ANOVA) for randomized block design followed by a Duncan's Multiple Range post-hoc test. Level of significance was declared at *p<*0.05. Pearson correlation coefficients were calculated with the aim to identify significant relationships (*p<*0.05) between nitrogen retention data, animal growth (PER), and PDCAAS. All statistical analyses were performed using the computer software Minitab16.

## Results

### Chemical composition of regular and soybean-fortified products

Formulation and chemical composition of the regular and soybean-fortified tortillas and yeast-leavened bread are summarized in Tables 1 and 2, respectively. The enriched cereal products containing 4.5 or 6.0% soybean concentrate or defatted soybean flour provided approximately 20% more protein, but similar amounts of energy compared to regular counterparts. The wheat flour tortillas contained higher calorie content (448.30 and 443.80 kcal/100 g) compared with maize tortillas (397.50 and 395.80 kcal/100 g). The addition of soybean protein to fortified foods increased the protein:calorie ratio compared to the respective regular counterparts (Table 2). It is worth mentioning that all experimental diets were formulated to contain equal amounts of protein, crude fat, crude fiber, and non-fibrous carbohydrates so the only sources of variation were the amino acid score and other not measured compounds present in soybeans like, for example, isoflavones (which were, however, not in the focus of this study).

**Table 2:** Chemical composition, caloric value, and amino acid composition of regular maize tortillas, wheat flour tortillas, and yeast-leavened bread foods fortified with soybean proteins^1^,^2^

	Maize tortillas		Yeast-leavened bread		Wheat flour tortillas	
			
	MT	SMT	BR	SBR	WT	SWT
Proximate composition, %						
Crude protein (N*6.25)	9.17±0.45	11.52±0.01	9.30±0.82	11.55±0.31	7.95±0.17	9.84±1.81
Crude fat	1.0±0.01	0.9±0.10	2.21±0.32	2.14±0.12	10.5±1.15	9.72±0.06
Crude fiber	1.7±0.14	2.0±0.07	0.4±0.28	0.5±1.84	0.2±1.41	0.3±0.49
Ash	0.8±0.02	0.9±0.02	1.4±0.10	1.4±0.11	1.2±0.05	1.3±0.07
Nitrogen-free extract^2^	87.33	84.68	86.69	84.41	80.15	78.84
Energy Value^3^ (kcal/100 g)	397.50	395.80	405.65	405.00	448.30	443.80
Protein:calorie ratio (g protein/100 Kcal)	2.30	2.91	2.29	2.85	1.77	2.21
Amino acids (g/100 g protein)						
Threonine	3.62	3.70	2.75	2.97	2.75	3.01
Valine	4.83	4.85	4.12	4.27	4.12	4.28
Isoleucine	3.62	3.89	3.61	3.84	3.61	3.83
Leucine	13.03	11.83	6.95	7.19	6.95	7.17
Lysine	2.90	3.73	2.32	3.23	2.32	3.19
Histidine	2.90	2.83	2.06	2.19	2.06	2.18
Tryptophan	0.72	0.93	1.37	1.38	1.37	1.42
Methionine + Cysteine	3.98	3.72	3.78	3.50	3.78	3.60
Phenylalanine + Tyrosine	7.60	7.88	7.12	7.52	7.12	7.46

1 MT, maize tortillas; SMT, soybean-fortified maize tortillas; BR, yeast-leavened bread; SBR, soybean-fortified bread; WT, wheat flour tortillas; SWT, soybean-fortified wheat tortillas. Values are means±SD and are expressed on dry matter basis, *n* = 3. The average moisture content of foods were: 51.79±1.25 for MT, 47.70±0.67 for SMT, 36.2±1.18 for BR, 37.30±1.43 for SBR, 29.04±0.82 for WT and 30.62±0.96 for SWT.

2 NFE = nitrogen-free extract that gives an indication of non-fibrous carbohydrates (starch plus sugars).

3 Calculated using the Atwater coefficient.

The amino acid compositions of the six foods are shown in Table 2. All fortified products were characterized by improved EAA composition especially in terms of lysine, tryptophan, and threonine. For instance, the most limiting amino acid lysine improved its concentration from 2.5 g/100 g protein (mean of lysine of MT, BR and WT) to around 3.4 g/100 g protein (mean of soybean-fortified counterparts). The regular and soybean-fortified maize tortillas contained 0.72 and 0.93 g of tryptophan (the second most limiting amino acid) per 100 g protein, respectively (Table 2). In this particular product, The amino acid score of lysine improved from 49 to 64 after soybean enrichment. Likewise, the EAA scores of soybean-fortified wheat flour tortillas and yeast-leavened bread improved in about 15 units (Table 3).

**Table 3:** Effect of soybean enrichment on amino acid scores of maize tortillas, yeast-leavened bread, and wheat flour tortillas foods^1^

		Maize tortilla		Yeast-leavened bread		Wheat flour tortilla	
				
Amino acids	Food agriculture Organization/World Health Organization Requirement 2–5 years infant	MT	SMT	BR	SBR	WT	SWT
Threonine	3.4	106	109	80	87	80	88
Valine	3.5	137	138	117	122	117	122
Isoleucine	2.8	129	138	128	137	128	136
Leucine	6.6	197	179	105	108	105	108
Lysine	5.8	**49**	**64**	**40**	**55**	**40**	**55**
Histidine	1.9	152	149	108	115	108	114
Tryptophan	1.1	65	84	124	125	124	129
Methionine + Cysteine	2.5	159	148	151	140	151	144
Phenylalanine + Tyrosine	6.3	120	125	113	119	113	118

1 MT, maize tortillas; SMT, soybean-fortified maize tortillas; BR, yeast-leavened bread; SBR, soybean-fortified bread; WT, wheat flour tortillas; SWT, soybean-fortified wheat tortillas. Amino acid scores calculated based on the Food and Agriculture Organization/World Health Organization requirement for 2–5 years infant. Values highlighted on bold letters depict the limiting amino acid.

### Animal growth performance (PER bioassay)

The results related to total diet intake and PER are summarized in Table 4. The daily food intake ranged from 6.23 to 11.52 g/d in groups of rats fed the experimental diets and averaged 13.41 g/d in the group fed the control casein diet (Table 4). Animals fed the soybean-fortified diets consumed significantly higher quantities of food (approximately 28% more) compared to their counterparts fed regular diets. Rats fed with soybean-fortified maize or wheat tortillas-based diets gained 1.86 and 2.28 g/day, respectively, while animals fed respective regular diets gained 0.47 and 0.59 g/day. Likewise, animals fed the soybean-fortified bread diet gained 0.74 g/day while their counterparts fed regular bread gained only 0.16 g/day. These differences in average daily gains were highly significant. Food intake, final body weight, daily weight gain, protein intake, and PER values were significantly higher (*p*<0.05) in casein and all soybean-fortified diets compared to the regular diets. The adjusted PER of the regular maize and wheat flour tortillas were 70 and 72 lower, respectively, compared to casein. On the other hand, the PER of these soybean-fortified products were 34 and 29 lower compared to casein.

**Table 4:** Effect of soybean protein enrichment of maize tortillas, yeast-leavened bread, and wheat flour tortillas on the growth and protein efficiency ratios (PER) of weanling rats fed formulated diets^1^,^2^

	Diets						
	
	Control casein	Maize tortillas		Yeast-leavened bread		Wheat flour tortillas	
				
	CSD	MTD	SMTD	BRD	SBRD	WTD	SWTD
Initial weight (g)	47.5±5.0 a	47.7±5.2 a	45.6±5.4 a	45.6±5.5 a	46.4±5.3 a	45.6±5.1 a	45.7±5.7 a
Final weight (g)	157.2±11.4 a	60.9±11.5 e	97.8±12.6 c	50.2±8.6 f	67.1±11.0 d	62.2±7.2 e	109.5±17.8 b
Average daily gain (g)	3.9±0.4 a	0.47±0.3 e	1.9±0.4 c	0.2±0.2 f	0.7±0.3 d	0.6±0.1 e	2.3±0.4 b
Food intake (g/day)	13.4±1.4 a	6.8±1.3 e	10.1±1.5 c	6.2±1.3 f	7.1±1.7 d	7.6±0.9 d	11.5±2.2 b
Protein intake (g/day)	1.2±0.1 a	0.5±0.1 f	0.9±0.1 c	0.6±0.1 e	0.6±0.2 d	0.7±0.1 d	1.0±0.2 b
Experimental PER	3.2±0.2 a	0.9±0.4 e	2.1±0.3 c	0.3±0.2 f	1.2±0.3 d	0.9±0.1 e	2.3±0.1 b
Adjusted PER	2.50 a	0.73 e	1.64 c	0.22 f	0.89 d	0.69 e	1.77 b
FER	0.29±0.11	0.07±0.08	0.19±0.02	0.03±0.03	0.10±0.03	0.08±0.01	0.20±0.01

1 MTD, maize tortillas; SMTD, soybean-fortified maize tortillas; BRD, yeast-leavened bread; SBRD, soybean-fortified bread; WTD, wheat flour tortillas; SWTD, soybean-fortified wheat tortillas; FER, Food Efficiency Ratio. Values are averages±SD of eight observations except for the control and regular maize tortillas treatments that experienced the death of one animal.

2 Means denoting different letter(s) within rows are statistically different (*p* < 0.05).

The average adjusted PER of the regular diets ranged from 0.2 to 0.7, whereas for soybean-fortified diets from 0.9 to 1.8. Therefore, the PERs of weanling rats fed with fortified diets were at least twice as high compared with animals fed their respective regular diets.

As shown in Table 4, animals fed the control casein diet had the highest adjusted PER. The adjusted PER values of the soybean-fortified maize and WTDs were 1.64 and 1.77, respectively (71 and 66% of the adjusted PER of the casein diet, respectively). The lowest PER values corresponded to rats fed the bread diets (regular and fortified).

### Nitrogen retention study

Dry matter and apparent protein digestibility, nitrogen retention values (BV and NPU), and PDCAAS are depicted in Table 5. As expected, animals fed with the casein-based diet had the highest dry matter and protein digestibility values and PDCAAS (97, 91, and 91% respectively).

**Table 5:** Effect of soybean protein enrichment of maize tortillas, yeast-leavened bread, and wheat flour tortillas on dry matter and protein digestibilities, nitrogen retention, and protein digestibility corrected amino acid scores (PDCAAS) estimated with weanling rats fed formulated diets^1^,^2^

	Diets						
	
	Control casein	Maize tortillas		Yeast-leavened bread		Wheat flour tortillas	
				
	CSD	MTD	SMTD	BRD	SBRD	WTD	SWTD
Dry matter digestibility (%)	96.5±0.4 a	89.3±2.4 d	90.8±0.8 d	94.6±0.9 b	94.6±1.9 b	90.6±0.1 d	92.3±0.5 c
Protein digestibility (%)	91.3±1.0 a	72.5±4.7 e	78.8±2.2 d	83.2±2.1 bc	84.8±2.2 b	81.4±2.8 c	83.0±1.6 bc
Biological value (%)	71.0±5.5 a	56.0±9.5 bc	72.4±13.1 a	44.5±17.1 d	52.2±11.2 c	57.9±8.6 bc	61.8±9.9 b
Net protein utilization value (%)	64.0±4.5 a	40.5±6.8 de	57.1±10.8 b	37.3±15.1 e	44.3±10.0 d	47.1±7.2 cd	51.4±8.3 bc
PDCAAS^3^	91.26	35.53	50.44	33.26	46.61	32.57	45.64

1 MTD, maize tortillas; SMTD, soybean-fortified maize tortillas; BRD, yeast-leavened bread; SBRD, soybean-fortified bread; WTD, wheat flour tortillas; SWTD, soybean-fortified wheat tortillas. Values are averages±SD of eight observations except for the control and regular maize tortillas treatments that experienced the death of one animal.

2 Means denoting different letter(s) within rows are statistically different (*p* < 0.05).

3 Protein digestibility corrected amino acid score. Calculated by multiplying protein digestibility by amino acid score (refer to Table 3).

The NPU and PER values were positively correlated with PDCAAS (*r=*0.85 and *r=*0.87, respectively, *p<*0.05). Therefore, all soybean-fortified products had higher nitrogen retention and PDCAAS values compared to their respective regular counterparts. The data clearly showed that the proposed soybean enrichment favored nitrogen retention estimated as BV and NPU; and animal growth estimated with the PER assay. Interestingly, the casein-based or the SMTDs showed similar BV (71 and 72%, respectively). The NPU value and PDCAAS scores of the fortified maize tortillas diet were 1.4 times higher compared to its regular counterpart. Among treatments, the BRD was with the lowest BV and NPU scores.

## Discussion

Results of this study clearly indicate that the proposed soybean enrichment intervention greatly improved EAA, PDCAAS, animal growth, and nitrogen retention values in a weanling rat model. According to the FAO, wheat and maize provided worldwide 526 and 146 kcal/day and 15.9 and 3.5 g/day protein in 2011, respectively (8). In Latin America, maize plays a more important role. For instance, maize, mainly in the form of tortillas and related products, provides 928 kcal/day and 26 g protein/day for the average Mexican.

The wheat flour tortillas contained the highest amount of energy due to the addition of shortening in the formulation. The ratio of protein to calories clearly favored the soybean-fortified products because of the protein enrichment (Table 2).

All fortified products were characterized by improved EAA composition especially in terms of lysine, tryptophan, and threonine because soybean is a rich source of these EAA. Soybean is especially rich in lysine and tryptophan. Defatted soybean flour containing around 50% protein provides approximately 10 times more lysine and tryptophan compared with regular maize tortilla flour. Therefore, the addition of only 6% soybean flour greatly improved the contents of these two limiting EAA. The same trend was observed with the lysine content of the wheat-based enriched products (Tables 2 and 3). The second limiting EAA in wheat flour tortillas and table bread was threonine. The enrichment with soybean proteins also slightly improved the amounts of this particular amino acid. The significant improvements in the EAA scores with similar levels of the soybean enrichment in maize tortillas, wheat flour tortillas, and table bread were previously documented by many authors (6, 7, 22–28).

Animals fed the casein- and soybean-fortified products consumed higher amounts of food compared with animals fed regular products due to the better EAA scores. Previous studies clearly indicated lower food intakes in animals consuming low-protein quality diets (6, 22, 24, 27, 28). The higher food intake plus the better EAA score of the soybean-fortified products improved caloric intake and greatly upgraded weight gains and PER (Table 4). An enhanced protein and energy intake would promote a faster recovery of children affected by protein calorie malnutrition, marasmus, or kwashiorkor. According to the WHO, a stronger formula that achieve energy and protein intake for catch-up growth in severe malnutrition patients should provide 100 kcal and 2.9 protein per 100 ml (29). All the soybean-fortified foods contained protein:calorie ratios varying from 2.2 to 2.9 g protein/100 kcal (Table 2), which are higher compared to the values ranging from 1.8 to 2.3, observed in regular counterparts. The improved protein calorie ratios of soybean-fortified foods were slightly lower or equal to the recommended values to combat protein-energy malnutrition (29). As expected, animals fed the casein control diet had the best protein digestibly, BV, NPU, PDCAAS, and PER values. Animals fed the soybean-fortified maize or wheat flour tortillas had about 30% less PER compared to counterparts fed the casein-based diet. This difference is attributed to a higher rate of protein digestibility (Table 5), and better EAA score and PDCAAS of casein-fortified compared with soybean-fortified products. However, from the practical point of view, casein costs about five times more than commercial defatted soybean flours used for the production of both types of tortillas and about twice as more compared to a soybean protein concentrate which was used for the production of enriched bread. Therefore, the intervention with soybean products is more recommended in terms of cost benefit.

Weanling rats fed with the soybean-fortified maize and soybean-fortified wheat tortilla diets gained 52.2 and 63.8 g during the 28-day growth trial compared with counterparts fed with regular products which gained 13.2 and 16.6 g, respectively. A similar trend in weight gain was evident in a study in which rats fed with fortified tortillas with 8% of defatted soybean meal and 4% of defatted sesame meal had weight gains 2.5 times higher compared with counterparts fed the control maize tortillas (28). Also Gonzalez Agramon and Serna Saldivar reported weight gains in weanling rats three times higher when they were fed with soybean-fortified wheat flour tortillas in comparison with animals fed regular wheat flour tortillas (23). These authors attributed the improved performance to the higher retention of nitrogen, which was mainly anabolized into body weight (23).

The higher PER values of the soybean-fortified maize and wheat tortillas were closer to the observed PER of animals fed the control diet. Previously, Chavez and Chavez reported that the PER of tortillas containing 6% of defatted soybean flour was 80% higher in comparison to the casein-based diet. In this study, the PER value of SMTD equaled 66% of PER value of casein (24). Moreover, weanling rats fed with soybean-fortified wheat flour tortillas had twice as high PER values when compared with rats fed with the unfortified tortillas (23). As shown in Table 4, rats fed the fortified wheat flour tortillas had average daily gains of 2.28 g while rats fed the regular WTD had an average daily gain of 0.59 g.

As shown in murine models, the proposed soybean enrichment of maize tortillas not only improves nitrogen retention and growth performance but also brain development of animals (6, 7, 25, 26).

Interestingly, animals fed the BRD diet were the ones that ended with the lowest gains and PERs. This occurred despite the fact that bread was fermented with dried yeast (2% based on flour weight), which is known to have adequate levels of all EAAs especially lysine (8 g/100 g of protein) (30). It is important to mention that among the cereal-based products tested herein, the bread formulation was the only food item containing supplemented sugar (5.5% based on flour weight) that is known to lower lysine bioavailability after thermal treatments such as the one applied during baking (17). Thus, the yeast-leavened bread-based diet was the treatment with the lowest BV and NPU scores. It is well known that sugars can interact with proteins and form Maillard compounds, interfering with lysine availability (31, 32). Apparently the level of heat treatment during baking probably affected amino acid bioavailability and protein digestibility (32, 33). There are some strategies to avoid the observed negative impact of sugar on protein quality. Prolonged fermentation time can lead to conversion of higher amounts of sugar into non-browning reaction metabolites. The other possible strategy is to employ leaner formulations in terms of supplemented sugars. French baguettes, pita, or Arabic flat breads and Chinese steamed breads, commonly produced with sugar-free formulations, should benefit more after soybean protein supplementation.

According to Mahmoodi et al., sugar negatively affected protein quality of soybean-fortified bread by decreasing the food efficiency ratio (FER) values estimated with rats (27). The 7% defatted soybean-fortified bread had a FER value of 0.18±0.01 whereas the same bread with 3% sugar had a significantly lower FER value (0.12±0.02). Concomitantly, in our study we found that bread supplemented with 4.5% soybean concentrate showed a FER value of 0.10±0.03 (Table 4) similar to Mahmoodi et al. (27).

The casein-based diet had the highest dry matter digestibility, apparent protein digestibility, and PDCAAS values, because casein possesses high digestibility and a complete EAA score, which increases nitrogen retention and, therefore, animal growth (23, 34).

It is important to mention that soybean products contain significant amounts of isoflavones that are known to prevent oxidative stress, chronic diseases, cancer and that possess weak estrogenic activity (35, 36). The defatted soybean flour and concentrate contain about 150 and 95 mg isoflavones/100 g, respectively (37). The isoflavone concentration in tortillas and bread is approximately 15 times lower because the wheat or nixtamalized flours were supplemented with 6% of defatted soybean flour or 4.5% of concentrate. Giampietro et al. concluded that soybean isoflavones did not significantly affect hormonal balance of children in a human intervention study (38).

## Conclusions

The addition of approximately 6% of defatted soybean flour to maize tortillas or wheat flour tortillas greatly improved the protein:calorie ratio, lysine and tryptophan contents, PER, nitrogen retention values, and PDCAAS. This protein quality improvement effect was not as evident in the yeast-leavened bread probably because its formulation contained sugar that upon baking reduced lysine bioavailability. Hence, intake of cereal-based soybean-fortified products, particularly those produced with sugar-free formulations, can benefit millions of people, especially children living in developing countries around the globe, who experience growth stunt due to the lack of good quality protein in their diets.
